# The General Acceptability and Use of Smartphone App-Delivered Interventions for Gambling in Australia

**DOI:** 10.1007/s10899-024-10373-9

**Published:** 2025-01-09

**Authors:** C. O. Hawker, S. S. Merkouris, A. C. Thomas, S. N. Rodda, S. Cowlishaw, N. A. Dowling

**Affiliations:** 1 School of Psychology, Deakin University, 221 Burwood Hwy, Burwood, VIC 3125 Australia; 2 Department of Psychology and Neuroscience, Auckland University of Technology, Auckland, 0627 New Zealand; 3 Turner Institute for Brain and Mental Health, Monash School of Psychological Sciences, Monash University, Clayton, VIC 3800 Australia

**Keywords:** Gambling, Smartphone, App, Internet, Intervention, Acceptability

## Abstract

Smartphones can extend the reach of evidence-based gambling treatment services, yet the general acceptability of app-delivered gambling interventions remains unknown. This study examined the general acceptability and use of app-delivered gambling interventions, and predictors of both, among 173 Australian adults with a lifetime gambling problem (48.5% male, M_age_ = 46.4 years) recruited from an online panel. Overall, 55.5% of the sample had a positive attitude toward app-delivered gambling interventions, 8.1% had a neutral attitude, and 36.4% had a negative attitude. Furthermore, one in five participants (20.8%) reported using an app-delivered gambling intervention in their lifetime. Four dimensions of acceptability were examined, wherein 78.6% of participants endorsed confidence in the effectiveness of app-delivered gambling interventions and 66.5% perceived anonymity benefits, while 48.6% endorsed scepticism (e.g., potential to increase isolation) and 69.4% perceived technology-related threats (e.g., difficulty learning and applying app-based strategies). Positive predictors of acceptability and use included younger age, rural/regional residence, gambling expenditure, problem gambling severity, gambling harms, and use of professional support. Acceptability did not increase the likelihood of using app-delivered gambling interventions, however, which may suggest a translation gap. The findings support continued investment into the development and evaluation of app-delivered gambling interventions, with a focus on enhancing engagement and uptake. Uptake could be improved by promoting the effectiveness and anonymity of evidence-based app-delivered gambling interventions, particularly among receptive audiences (young people, rural/regional residents, those with greater problem gambling severity), while redressing scepticism and perceived technology-related threats among vulnerable subgroups (those with greater gambling expenditure and gambling-related harm).

**Supplementary Information:**

The online version contains supplementary material available at 10.1007/s10899-024-10373-9.

## Introduction

Gambling harms pose a significant threat to the “health, financial security, and social wellbeing of millions of people worldwide” (Price et al., 2021; p.37). While people with problem gambling^1^ typically experience the most severe gambling harms (Browne et al., 2016), people across the continuum of risk report far-reaching negative consequences for their finances, relationships, health, occupations, connection to culture, and legal status (Langham et al., 2016). Moreover, these negative consequences extend beyond the individuals who gamble, and impact their families and broader communities (Langham et al., 2016; Price et al., 2021). Despite facing a high burden of harm (Browne et al., 2016), few people experiencing gambling problems seek any help (1 in 5 people with severe gambling problems, and 1 in 25 with moderate-risk gambling problems; Bijker et al., 2022) due to individual and structural barriers, which can hinder early intervention and treatment efforts (Khayyat-Abuaita et al., 2015; Loy et al., 2019).

Smartphone apps offer opportunities to extend the reach of evidence-based interventions, including cognitive behaviour therapy, motivational interviewing, and third-wave approaches (e.g., mindfulness) to people experiencing problems with their gambling (Cowlishaw et al., 2012; Di Nicola et al., 2020; Maynard et al., 2018; Pfund et al., 2023; Thomas et al., 2011; Yakovenko et al., 2015). Hence, psychological interventions delivered via smartphone apps have attracted mounting research attention given their ability to overcome numerous barriers to accessing in-person treatment (McCurdy et al., 2023). First and foremost, smartphone apps are highly portable, convenient, and offer unprecedented access to therapeutic content, potentially anywhere and anytime (Bakker et al., 2016; Carpenter et al., 2020; Klasnja & Pratt, 2012). Smartphone apps are cost-effective, as many are free or low-cost in contrast to oftentimes expensive in-person services (Bakker et al., 2016; Klasnja & Pratt, 2012). Smartphone apps provide low-burden support and offer anonymity (Bakker et al., 2016; Carpenter et al., 2020; Klasnja & Pratt, 2012), which can overcome some of the individual barriers faced by people experiencing gambling problems, particularly low motivation, shame, and stigma (Dąbrowska et al., 2017; Gainsbury et al., 2014). Furthermore, smartphones have a global penetration rate of roughly 70% (Statista, 2023), rendering them a highly scalable modality for psychological interventions, as well as a conduit for delivering evidence-based interventions to underserved populations, such as those in rural and regional areas who often face geographical barriers to accessing in-person services (Dąbrowska et al., 2017; Gainsbury et al., 2014).

While smartphone apps can provide highly accessible treatment for people experiencing gambling problems, the field has moved slowly (Brownlow, 2021). Despite high market demand, two recent reviews of publicly available apps aimed at managing problematic gambling behaviour have underscored a paucity of apps that are evidence-based and theoretically grounded (McCurdy et al., 2023; Ridley et al., 2020). For example, only a minority of such apps available in Australia were affiliated with an established addition service (14%; 6 of 42 apps) or utilised a recognisable treatment model (24%; 10 of 42 apps; Ridley et al., 2020). Furthermore, only a small number of apps developed to deliver evidence-based psychological interventions for gambling have been evaluated in the literature. Four such apps were derived from the *GamblingLess* evidence-based online intervention for gambling (Dowling et al., 2021), including an app targeting gambling cravings (Hawker et al., 2021; Merkouris et al., 2020), an app targeting the cognitive processes underpinning relapse (gambling cravings, self-efficacy, and positive outcome expectancies; Dowling 2024a, 2024b; Dowling et al., 2022), an app providing support to implement gambling expenditure limits (Rodda et al., 2022, 2024), and a culturally responsive app for people in Aotearoa New Zealand (Humphrey et al., 2020, 2022). In addition, So and colleagues (2024; 2020) evaluated a ‘chatbot’ app that delivered monitoring, feedback, and therapeutic content for gambling. The collective results from single-arm trials (Hawker et al., 2021), randomised controlled trials (Humphrey et al., 2020; So et al., 2024), micro-randomised trials (Dowling et al., 2024a; Rodda et al., 2024a), and qualitative evaluations (Humphrey et al., 2022) of these gambling app interventions showed improvements in problem gambling severity, gambling symptom severity, gambling behaviour, gambling cravings, strength of intention to adhere to gambling goals, self-efficacy, and outcome expectancies.

As the field of app-delivered gambling interventions is in its formative stages, studies have emphasised the importance of examining app acceptability and effectiveness prior to implementation and dissemination (e.g., Ebert et al., 2018; Gun et al., 2011; Merkouris et al., 2020). The acceptability of the aforementioned gambling app interventions has been measured in various ways, such as via visual analogue scales of helpfulness, burdensomeness, and relevance (Dowling et al., 2024b; Hawker et al., 2021; Rodda et al., 2024b), items measuring user satisfaction and the likelihood of recommending the app to others (Dowling et al., 2024b; Hawker et al., 2021; Rodda et al., 2024b; So et al., 2020), app usage and engagement indices (Dowling et al., 2024b; Hawker et al., 2021; Rodda et al., 2024b; So et al., 2020), and semi-structured interviews (Dowling et al., 2024b; Rodda et al., 2024b). Acceptability was measured favourably across all of these studies, except for participants’ likelihood of recommending the ‘chatbot’ intervention (So et al., 2020), where the majority of participants rated the likelihood as a 6 or below out of 10 (considered a negative score). Despite overall positive reports of the acceptability of these gambling app interventions, researchers have argued that evaluations of the acceptability of interventions delivered using technology, such as smartphone apps and the internet, are inadequate and lacking uniformity (Schröder et al., 2015; Sekhon et al., 2017). Indeed, Schröder and colleagues (2015) illustrate that the acceptability of such interventions has been measured via blunt single item measures, retrospective reports that ignore general attitudes, or inferences based on potentially biased ‘completers-only’ data.

To redress the argued shortcomings of the measurement of the acceptability of interventions delivered using technology, Schröder and colleagues developed the Attitudes towards Psychological Online Interventions Questionnaire (APOI) as a purpose-built measure of acceptability, which was defined as a “cognitively based, positive attitude towards such interventions” (Schröder et al., 2015; p.137). A four-factor model was retained in the final exploratory factor analysis, including: (1) *Confidence in Effectiveness* (e.g., expectations that a psychological online intervention [POI] can help to identify issues to challenge); (2) *Anonymity Benefits* (e.g., perception of POIs as more confidential and discreet than seeing a therapist); (3) *Scepticism and Risk Perception* (e.g., scepticism about a POI delivering professional support and potentially increasing risk of isolation); and (4) *Technology-Related Threats* (e.g., concerns about the likelihood of learning skills to manage everyday life and stay motivated when using a POI compared to a therapist). This model was found to represent the dimensionality of attitudes toward POIs, which could also be meaningfully interpreted as an overall attitude indicative of general acceptability.

As the only validated measure of its type, the APOI has been increasingly employed to measure the acceptability of various online psychological interventions utilised by clinical (e.g., Jelinek et al., 2023; Schröder et al., 2017, 2020; Westermann et al., 2020) and community samples (e.g., Molloy et al., 2021; Ramos et al., 2022). Furthermore, the APOI has been adapted to examine the acceptability of smartphone app-delivered psychological interventions. Bruhns and colleagues (2023; 2021) examined the acceptability and effectiveness of a transdiagnostic self-help app (“MCT & More”) designed to reduce depressive symptoms. In a sample of 400 students with depressive symptoms who utilised the app (Bruhns et al., 2021), 87.3% endorsed confidence in its effectiveness and 45.5% perceived anonymity benefits, while 31.8% endorsed scepticism and perceived risks and 67.8% perceived technology-related threats. Similar results were found in a later study, involving 159 inpatients with depression who utilised the app after discharging from a psychiatric hospital (Bruhns et al., 2023), whom reported an overall positive attitude toward smartphone app-delivered interventions. Both studies also found that overall positive attitudes were positively correlated with the frequency of using the app. No studies, however, have examined the general acceptability of app-delivered gambling interventions utilising a validated measure, such as the APOI.

Given that governments around the world are investing significant resources into the development and integration of technology into the delivery of evidence-based healthcare treatment (Australian Digital Health Agency, 2022; Wilson et al., 2023), including gambling treatment (McCurdy et al., 2023), research is required to examine the general acceptability of app-delivered gambling interventions. Such examinations may shed light on the potential tailoring of intervention delivery toward harder-to-reach populations (e.g., rural/regional residents) and receptive audiences. For example, predictors of app acceptability in previous research have included younger age, existing use of treatment services, and greater or more severe mental health-related symptomology (e.g., Aziz et al., 2023; Lipschitz et al., 2020; Smail-Crevier et al., 2019). Furthermore, such examinations may reveal particular concerns associated with app-delivered gambling interventions among people experiencing gambling-related harm. Taken together, examinations of the acceptability of app-delivered gambling interventions may support efforts to increase their reach, uptake, and effectiveness, to ultimately improve intervention efforts addressing the high global burden of harm associated with gambling (Price et al., 2021).

Accordingly, this study aimed to explore the general acceptability and use of smartphone app-delivered gambling interventions, and to identify predictors of acceptability and use, in a sample of Australian adults with gambling problems. It was hypothesised that people who were young, male, rural/regional residents, and who displayed greater gambling behaviour (frequency and expenditure), problem gambling severity, gambling harms, and use of gambling support would endorse greater acceptability and use of app-delivered gambling interventions.

## Method

### Participants

The sample comprised 173 adults who positively endorsed the One-Item Screen (Dowling et al., 2019; Thomas et al., 2010), in which respondents self-identify as “ever having an issue with their gambling” (i.e., a lifetime gambling problem). Participants were recruited in July 2023 via Qualtrics, an online panel provider, to take part in a broader study that also explored the awareness and acceptability of a local app-delivered gambling intervention (“Reset”; reported elsewhere: Dowling et al., 2024a, b, c). Respondents were eligible to participate if they were 18 years old or above, lived in Victoria (Australia), and endorsed having a lifetime gambling problem. This study had ethics approval (Deakin University Human Research Ethics Committee: reference 2021–398).

### Measures

The survey comprised measures of the general acceptability and use of app-delivered gambling interventions, as well as potential predictors of acceptability and use (demographic characteristics, gambling behaviour, problem gambling severity, and gambling harms).

**Demographic characteristics**. Participants were asked to report their age (in years), gender, and residential postcode. Postcodes were categorised as metropolitan or rural/regional using publicly available postal data (Department of Home Affairs, 2022).

**Gambling behaviour**. Participants were asked to report their past-year gambling frequency (number of times) on eleven gambling activities (e.g., lotteries, sports betting, etc.), as well as their gambling expenditure ($AUD lost) on any activity with a reported frequency of one or more. Gambling frequency and expenditure were aggregated across the eleven gambling activities; where gambling participation was indicated by an aggregated frequency of one or more.

**Problem gambling severity**. The nine-item Problem Gambling Severity Index (PGSI: Ferris & Wynne, 2001) was used to measure characteristics of problem gambling behaviour (e.g., betting more than one can afford) and adverse consequences from gambling (e.g., financial problems) experienced over the past year on a 4-point scale from 0 (*never*) to 3 (*almost always*). Total scores indicate non-problem gambling (score of 0), low-risk gambling (scores of 1–2), moderate-risk gambling (scores of 3–7), and problem gambling (scores of 8–27). The PGSI is a frequently used and validated measure (Currie et al., 2013), which displayed excellent internal consistency in this study (Cronbach’s alpha = 0.95).

**Gambling harms**. The seven-item Domain-General Gambling Harm Scale (DGHS-7: Syvertsen et al., 2023) was used to measure gambling harms experienced over the past year across seven established harm domains (Langham et al., 2016). Participants were asked to report the level of negative impact of their gambling in the financial, relationship, emotional/psychological wellbeing, physical/mental health, work/study performance, cultural/religious, and law-abidingness domains on a 5-point scale from 0 (*no impact*) to 4 (*major impact*). Higher scores indicate a greater degree of harm. The DGHS-7 is a newly developed and validated measure, which has displayed strong correlations with other validated measures of gambling harm (e.g., the 72-item harms checklist, r = 0.824; Li et al., 2017), and excellent internal consistency in this study (Cronbach’s alpha = 0.94).

**Professional help-seeking**. An adapted version of the Help-Seeking Questionnaire (Rodda et al., 2018) was employed to measure the lifetime use of eight professional gambling support options on a yes/no scale, including five ‘high-intensity’ options (e.g., saw a gambling counsellor in-person) and three ‘low-intensity’ options (e.g., emailed a gambling counsellor).

**General acceptability of app-delivered gambling interventions**. The APOI (Schröder et al., 2015) was adapted to measure the general acceptability of app-delivered gambling interventions. Each item substituted the term “psychological online intervention” with “gambling help app” (defined as apps that aim to help people manage their gambling). Participants rated their agreement on a 5-point scale with 16 statements that evenly span four subscales to measure ‘confidence in the effectiveness’, ‘anonymity benefits’, ‘scepticism’, and ‘technology-related threats’ in relation to app-delivered gambling interventions (description of items provided in Table 3). Positively-valenced subscales were reverse scored; hence, higher subscale scores (range: 4–20) and total scores (range: 16–80) indicated greater acceptability. Scores were categorised into “positive attitude” (total score ≥ 49; subscale score ≥ 13, i.e., higher confidence in effectiveness/anonymity benefits, and lower scepticism/technology-related threats), “neutral attitude” (total score of 48; subscale score of 12), or “negative attitude” (total score ≤ 47; subscale score ≤ 11, i.e., lower confidence in effectiveness/anonymity benefits, and higher scepticism/technology-related threats) (Bruhns et al., 2021).

The APOI was validated in a multi-phase study (validation phase: *n* = 305; Schröder et al., 2015). The results of a confirmatory factor analysis demonstrated that all items loaded significantly on their respective first-order factors, inter-correlations among the subscales were small-to-moderate and reasonable, and internal consistency was acceptable-to-good (Cronbach’s alpha: 0.77 [total APOI score]; range: 0.62–0.74 [across the APOI subscales]). In the current study, the APOI subscales also displayed acceptable-to-good internal consistency (Cronbach’s alpha: 0.72–0.84).

**Use of app-delivered gambling interventions.** Participants were asked whether they had ever used an app-delivered gambling intervention via three single items that specifically asked about the lifetime use of two Australian apps (“Reset”, “100-Day Challenge”) and “any other” app-delivered gambling intervention on a yes/no scale. Lifetime use of an app-delivered gambling intervention was indicated by endorsement of any of the three items.

### Procedure

The online panel provider (Qualtrics) hosted the survey and recruited the sample to participate in a broader study that explored the awareness and acceptability of a local app-delivered gambling intervention. Qualtrics only advertise the study duration (15 min) and varying reimbursements (independently offered by the panel provider, such as gift cards, redeemable points, etc.) to potential survey respondents registered on their databases. Potential respondents can opt to visit the survey landing page, which outlined the aims of the study, obtained informed consent to participate, and administered three items to confirm eligibility to participate (i.e., age over 18 years, Victorian residential postcode, endorsement of a lifetime gambling issue). Qualtrics ran duplication and validation checks to remove participants who straight-lined through the survey responses (*n* = 5) or provided poor quality open-ended responses, including non-insightful (*n* = 15), overly similar (*n* = 12), or gibberish (*n* = 1) responses across multiple questions, until the target sample of a minimum of 170 participants was achieved. Green’s rule of thumb for a medium effect of testing coefficients within a regression model determined that a sample of *n* = 105 was sufficient for inferential analyses involving a single independent variable; notably, *n* = 170 allowed for a maximum of 15 independent variables in a multiple regression (Green, 1991 as cited in VanVoorhis & Morgan, 2007).

### Data Analysis

Statistical analyses were conducted using Stata v18.0 (StataCorp, 2023). There was no missing data due to forced item responses. The gambling frequency and expenditure variables were non-normally distributed and trimmed to remove two extreme outliers that likely indicated poor data quality (e.g., lottery expenditure of $1.000e + 32) (Tabachnick & Fidell, 2019). Descriptive statistics were calculated, including means and standard deviations for continuous variables (except median and interquartile ranges for the non-normally distributed gambling behaviour variables) and count and percentages for categorical data.

A series of regressions were employed to explore whether demographic and gambling-related variables predicted APOI subscale scores (via linear regressions) and the lifetime use of app-delivered gambling interventions (via binary logistic regressions), and whether the APOI subscales predicted app use. PGSI and DGHS-7 total scores were treated continuously in the regression analyses. Robust estimators were utilised where regressions involved the APOI “Confidence in Effectiveness” subscale due to its non-normal distribution. Variables with significant associations in univariate regressions were explored further in multivariate regressions. While multicollinearity checks indicated VIF estimates below five, post hoc testing was conducted on the PGSI and DGHS-7 due to moderate VIF values (3.79 and 4.45, respectively), which revealed a very strong correlation between the two variables (r = 0.84). Consequently, DGHS-7 gambling harms was removed from multivariate regressions involving PGSI problem gambling severity to improve the predictive power of the model (Paul, 2006). The magnitude of effect sizes was considered small (OR = 1.68; β = 0.10–0.29), medium (OR = 3.47; β = 0.30–049), or large (OR = 6.71; β ≥ 0.50) (Chen et al., 2010; Cohen, 1988). Significance was set at *p* = 0.05 for all analyses.

## Results

### Descriptive Statistics

Table 1 displays the sample descriptive statistics. The sample comprised 173 adults (48.5% male) with a lifetime gambling problem, who had a mean age of 46.4 years (SD = 18.0) and mostly lived in a metropolitan area (81.5%). Almost the entire sample gambled in the past year (97.7%), most often on lotteries (79.2%), electronic gaming machines (61.3%), and horse/race betting (59.5%). On average, participants reported gambling nearly once a week and spending nearly AUD$800 in the past year. The mean PGSI score (8.48, SD = 7.53) was classified in the problem gambling category, with 91.3% of the sample displaying some level of gambling problems over the past year. Specifically, almost half of the sample displayed past-year problem gambling (45.1%), compared to 27.8% with moderate-risk gambling, 18.5% with low-risk gambling, and 9.7% with non-problem gambling. DGHS-7 scores indicated that 87.3% of the sample endorsed experiencing gambling harms in the past year, with financial (80.4%), emotional/psychological wellbeing (75.1%), and physical/mental health (67.6%) harms the most common. Approximately one-third of the sample reported ever using high-intensity (33.5%) or low-intensity (28.9%) professional gambling support (combined total of 39.3%).

**Table 1:** Demographic and gambling-related characteristics for the study sample (n = 173)

	Total (n = 173)
**Demographic characteristics**	
*Age, categories (n, %)*	
18–29 years	40 (23.12)
30–39 years	34 (19.65)
40–49 years	21 (12.14)
50–59 years	35 (20.23)
60–69 years	20 (11.56)
70 + years	23 (13.29)
*Gender (n, %)*	
Male	84 (48.55)
Female	89 (51.45)
*Residential area (n, %)*	
Metropolitan	141 (81.50)
Rural/regional	32 (18.50)
**Gambling-related characteristics**	
*Past-year gambling participation (n, %)*	169 (97.69)
Lotteries	137 (79.19)
Electronic gaming machines (EGMs)	106 (61.27)^a^
Betting on horse or harness racing or greyhounds	103 (59.54)
Betting on sports (not including sweeps, fantasy sports, eSports)	86 (49.71)
Casino table games	51 (29.48)
Bingo	50 (28.90)
Keno	39 (22.54)
Scratch tickets	38 (21.97)
eSports (e.g., video game tournaments)	35 (20.23)
Fantasy sports (e.g., “fantasy” leagues of real athletes)	23 (13.29)
Other gambling activity (e.g., private gambling, sweeps, competitions)	50 (28.90)
*Past-year overall gambling behaviour (median, IQR)*	
Gambling frequency (number of times)	48 (98.50 [117.00–18.50)
Gambling expenditure (money lost; $AUD)	$780 ($2,845 [$3,110.00–$255.00)
*Past-year PGSI problem gambling category (n, %)*	
Problem gambling	78 (45.09)
Moderate-risk gambling	48 (27.75)
Low-risk gambling	32 (18.50)
Non-problem gambling	15 (8.67)
*Past-year DGHS-7 gambling harms (n, %)*^*b*^	
Any harms	151 (87.28)
Financial harms	139 (80.35)
Emotional/psychological wellbeing harms	130 (75.14)
Physical/mental health harms	117 (67.63)
Relationship harms	96 (55.49)
Work/study performance harms	94 (54.34)
Cultural/religious harms	72 (41.62)
Law-abidingness	64 (36.99)
*Lifetime professional help-seeking (n, %)*	
High-intensity options	58 (33.53)
Low-intensity options	50 (28.90)
High- or low-intensity options	68 (39.31)

*M* Mean, *SD* standard deviation, *IQR* interquartile range, *PGSI* Problem Gambling Severity Index, *DGHS-7* Domain-General Gambling Harm Scale-7, Median and IQR (including calculation to obtain IQR due to wide ranges) reported for overall gambling frequency and expenditure due to extreme outliers in the data. Residential area was classified as metro or rural/regional based on postcode data

^a^Based on n = 172 due to removal of an extreme outlier for EGM frequency, which was used to calculate EGM participation

^b^Gambling harms were categorised using a DGHS-7 item cut-score of 1 (“minor impact”)

### General Acceptability and Use of App-Delivered Gambling Interventions

Table 2 and 3 display descriptive statistics of the dependent variables and the APOI items, respectively. The mean APOI total score was 50.0 out of 80 (SD = 8.5) and mean item scores ranged from 2.27 to 3.80 out of 5. Of the 173 participants, 96 (55.5%) had a positive attitude toward app-delivered gambling interventions, 14 (8.1%) had a neutral attitude, and 63 (36.4%) had a negative attitude. The majority of participants endorsed: (1) confidence in the effectiveness (78.6%), particularly for increasing recognition of issues to challenge; and (2) perceived anonymity benefits (66.5%), particularly for increasing the ease with which feelings can be divulged compared to therapist-delivered treatment. On the other hand: (3) almost half of the sample endorsed scepticism (48.6%), particularly about the provision of professional support via an app-delivered intervention; and (4) the majority of participants (69.4%) perceived technology-related threats, particularly in relation to potential difficulty understanding therapeutic concepts compared to therapist-delivered treatment. Overall, a fifth of the sample (*n* = 36; 20.8%) reported lifetime use of an app-delivered gambling intervention.

**Table 2:** Acceptability and use of app-delivered gambling interventions among people with a lifetime gambling problem (n = 173)

Acceptability and use of app-delivered gambling interventions	Total (n = 173)
*Total APOI acceptability (M, SD)*^*a*^	50.04 (8.47)
Confidence in effectiveness subscale^b^	14.85 (3.22)
Anonymity benefits subscale^b^	13.91 (3.37)
Scepticism and perception of risks subscale^b^	11.51 (3.22)
Technology-related threat subscale^b^	9.76 (3.08)
*Total APOI acceptability (n, %)*^*c*^	
Positive attitude	96 (55.49)
Neutral attitude	14 (8.09)
Negative attitude	63 (36.42)
*Confidence in effectiveness subscale*	
Positive attitude (i.e., higher confidence)	136 (78.61)
Neutral attitude	15 (8.67)
Negative attitude (i.e., lower confidence)	22 (12.72)
*Anonymity benefits subscale*	
Positive attitude (i.e., higher anonymity benefits)	115 (66.47)
Neutral attitude	19 (10.98)
Negative attitude (i.e., lower anonymity benefits)	39 (22.54)
*Scepticism and perception of risks subscale*	
Positive attitude (i.e., lower scepticism)	59 (34.10)
Neutral attitude	30 (17.34)
Negative attitude (i.e., higher scepticism)	84 (48.55)
*Technology-related threats subscale*	
Positive attitude (i.e., lower threat)	33 (19.08)
Neutral attitude	20 (11.56)
Negative attitude (i.e., higher threat)	120 (69.36)
*Lifetime use of app-delivered gambling interventions (n, %)*	36 (20.81)
“100-Day Challenge” app	21 (12.14)
“Reset” app	12 (6.94)
Other	22 (12.72)

*APOI* Attitudes toward psychological online interventions (adapted for app-delivered gambling interventions)

^a^APOI total scores range from 16 to 80

^b^APOI subscale scores range from 4 to 20, where higher scores indicate greater acceptability

APOI scores were categorised into “positive attitude” (total score ≥ 49; subscale score ≥ 13), “neutral attitude” (total score of 48; subscale score of 12), or “negative attitude” (total score ≤ 47; subscale score ≤ 11; Bruhns et al., 2021)

**Table 3:** Mean and standard deviations for APOI items (n = 173)

	M (SD)
*Confidence in effectiveness*	
Help to increase recognition of issues to challenge	3.80 (0.95)
General helpfulness	3.56 (1.05)
Provision of inspiration to better approach problems	3.75 (0.95)
Concept makes sense	3.74 (0.99)
*Anonymity benefits*	
Confidentiality and discretion compared to therapist-delivered treatment	3.60 (1.07)
Ease of revealing feelings compared to a therapist	3.62 (1.04)
Likelihood of telling friends compared to therapist-delivered treatment	3.20 (1.17)
No fear that someone will find out about gambling problem	3.49 (1.11)
*Scepticism and perception of risks*	
Expectations of long-term effectiveness	2.83 (1.07)
Provision of professional support	3.07 (1.10)
Ability to effectively implement strategies in everyday life	2.66 (1.05)
Potential to increase isolation and loneliness	2.95 (1.15)
*Technology-related Threats*	
Help in crisis situations compared to a therapist	2.27 (0.97)
Ability to learn skills to manage everyday life compared to therapist-delivered treatment	2.44 (0.97)
Likelihood of staying motivated compared to therapist-delivered treatment	2.39 (0.99)
Understanding of therapeutic concepts compared to therapist-delivered treatment	2.66 (1.00)

Items were scored on a scale from 1 “totally agree” to 5 “totally disagree”; item scores were reversed on the Confidence in Effectiveness and the Anonymity Benefits subscales. Higher mean scores (range: 0–5) indicate greater acceptability of app-delivered gambling interventions (hence, higher scores on the Scepticism and Perception of Risks and Technology-related Threats subscales indicate lower scepticism and lower threat, respectively)

### Predictors of the Acceptability and Use of App-Delivered Gambling Interventions

The results of the univariate and subsequent multivariate regressions are displayed in Tables 4 and S1 (supplementary material), respectively. A series of univariate linear regressions revealed the following significant associations with small effect sizes: (1) Greater problem gambling severity was associated with higher anonymity benefits; (2) Greater gambling expenditure and gambling harms were associated with higher scepticism and perception of risks; and (3) Rural/regional residence was associated with lower technology-related threats, whereas greater use of high-intensity support options was associated with higher technology-related threats. There were no significant predictors of Confidence in Effectiveness subscale scores. Multivariate analyses identified that: gambling harms independently predicted scepticism (B = − 0.016, SE = 0.03, 95%CI − 0.12, − 0.01, β = − 0.17, *p* = 0.033); and rural/regional residence (B = 1.56, robust SE = 0.59, 95%CI 0.39, 2.72, β = 0.20, *p* = 0.009) and use of high-intensity support options (B = − 0.35, robust SE = 0.17, 95%CI − 0.68, − 0.03, β = − 0.16, *p* = 0.035) independently predicted technology-related threats.

**Table 4:** Univariate regression analyses of the relationship between the demographic and gambling-related characteristics with the acceptability and lifetime use of app-delivered gambling interventions (n = 173)

	B (95% CI)				OR (95%CI)
	Confidence in effectiveness^a^	Anonymity benefits	Scepticism and Risk Perception	Technology-related Threat	Use of app-delivered gambling interventions
Age (years)	− 0.01 (− 0.04, 0.02) β = − 0.05	− 0.01 **(**− 0.03, 0.02) β = − 0.03	0.01 (− 0.02, 0.04) β = 0.05	− 0.00 **(**− 0.03, 0.03) β = − 0.00	0.97 (0.95, 0.99)*
Male gender	0.38** (**− 0.59, 1.35) β = 0.06	0.80 **(**− 0.20, 1.81) β = 0.12	0.38 (− 0.59, 1.34) β = 0.06	0.10 (− 0.88, 0.98) β = 0.01	1.23 (0.59, 2.58)
Rural/regional residence	0.18** (**− 1.00, 1.37) β = 0.02	0.14 **(**− 1.16, 1.45) β = 0.02	0.44 (− 0.80, 1.69) β = 0.05	1.56 (0.39, 2.73)** **β = 0.20**	1.65 (0.69, 3.97)
High-intensity support	0.24 **(**− 0.10, 0.58) β = 0.10	0.06 **(**− 0.31, 0.44) β = 0.03	− 0.22 (− 0.58, 0.13) β = − 0.09	− 0.36 (− 0.70, − 0.03)* **β = − 0.16**	2.94 (2.09, 4.14)***
Low-intensity support	0.27** (**− 0.28, 0.82) β = 0.08	0.13 **(**− 0.44, 0.70) β = 0.03	− 0.35 (− 0.89, 0.19) β = − 0.10	− 0.34 **(**− 0.85, 0.18) β = − 0.10	5.87 (3.45, 10.00)***
Gambling frequency^b^	0.00** (**− 0.00, 0.00) β = 0.03	0.00 **(**− 0.00, 0.00) β = 0.05	− 0.00 (− 0.00, 0.00) β = − 0.06	− 0.00 **(**− 0.00, 0.00) β = − 0.01	1.00 (0.99, 1.00)
Gambling expenditure^b^	0.00** (**− 0.00, 0.00) β = 0.04	0.00 **(**− 0.00, 0.00) β = 0.06	**− 0.00** (− 0.00, − 0.00)* **β = − 0.15**	− 0.00 (− 0.00, 0.00) β = − 0.11	1.00 (0.99, 1.00)
Problem gambling severity	0.04** (**− 0.03, 0.11) β = 0.09	0.08 (0.01, 0.15)* **β = 0.18**	− 0.06 (− 0.12, 0.01) β = − 0.14	− 0.05 **(**− 0.11, 0.01) β = − 0.12	1.13 (1.07, 1.19)***
Gambling harms	0.1321** (**− 0.0517, 0.0860) β = 0.049	0.0642 (− 0.004, 0.1279)* β = 0.147	− 0.07 (− 0.13, − 0.01)* **β = − 0.18**	− 0.05 **(**− 0.10, 0.00) β = − 0.14	1.17 (1.11, 1.24)***
Confidence in Effectiveness^a^	–	–	–	–	1.08 (0.96, 1.22)
Anonymity Benefits	–	–	–	–	1.06 (0.95, 1.19)
Scepticism and Risk Perception	–	–	–	–	1.02 (0.91, 1.14)
Technology-related Threat			–	–	1.03 (0.91, 1.16)

Bolded text indicates a significant result

Linear regressions were utilised to explore the acceptability of app-delivered gambling interventions and binary logistic regressions were utilised to explore the lifetime use of app-delivered gambling interventions

*B* unstandardised beta, *β* standardised beta, *95% CI* 95% confidence interval, *OR* odds ratio

^a^Robust estimators were used due to non-normally distributed data and/or extreme outliers

^b^n = 172 due to trimmed data to deal with extreme outliers on the gambling expenditure variable

Variables that were significant at p < 0.05 in univariate analyses were explored further in multivariate analyses. Higher APOI scores indicate greater acceptability of app-delivered gambling interventions (hence, higher scores on the Scepticism and Perception of Risks and Technology-related Threat subscales indicate lower scepticism and lower threat, respectively)

A series of univariate binary logistic regression analyses revealed that young people and people with higher use of low-intensity and high-intensity supports, greater problem gambling severity, and greater gambling harms had higher odds of app-delivered gambling intervention use; where all associations had small effect sizes, except for a large association between the use of low-intensity support and an app-delivered gambling intervention. In contrast, none of the APOI subscales predicted app use. Multivariate analyses identified that the use of high-intensity (OR = 1.82, SE = 0.38, 95%CI 1.21, 2.73, *p* = 0.004) and low-intensity (OR = 3.49, SE = 1.09, 95%CI: 1.89, 6.44, *p* < 0.000) support options independently increased the odds of using app-delivered gambling interventions.

## Discussion

This study is the first to examine the general acceptability and use of smartphone app-delivered psychological interventions for gambling, as well as to identify the predictors of acceptability and use, among people with lifetime gambling problems. Acceptability was defined as a cognitively-based positive attitude toward app-delivered interventions (Schröder et al., 2015), with the study results indicating that the majority of the sample (55.5%) displayed a positive attitude toward app-delivered gambling interventions, compared to 8.1% who displayed a neutral attitude and 36.4% who displayed a negative attitude.

### Confidence in the Effectiveness and Perceived Anonymity Benefits

This study utilised an adapted version of a validated measure of general acceptability (Schröder et al., 2015), which examined two positively-framed dimensions of: (1) confidence in the effectiveness of app-delivered gambling interventions to support the recognition of issues to challenge, be generally helpful, provide inspiration to approach problems, or be a logical concept; and (2) perceived benefits of anonymity for the confidentiality and discretion, ease of revealing feelings, likelihood of telling friends about using an app, or lack of fear about others finding out about a gambling problem.

Overall, the majority of the sample endorsed confidence in the effectiveness (78.6%) and anonymity benefits (66.5%) of app-delivered gambling interventions. In addition, greater problem gambling severity significantly predicted greater perceived benefits of anonymity. This finding suggests that app-delivered gambling interventions have the potential to overcome some of the individual barriers of shame, stigma, and a desire to self-manage gambling problems (Dąbrowska et al., 2017; Gainsbury et al., 2014) among people with more severe gambling problems. Given that problem gambling is “among the most stigmatised mental health problems” (Quigley et al., 2020; p.410), this finding holds promise for the capacity of app-delivered interventions to extend the reach of evidence-based gambling treatment. Unexpectedly, none of the demographic or gambling-related characteristics significantly predicted confidence in the effectiveness of app-delivered gambling interventions, which suggests that such confidence was robust across a diverse range of people with gambling problems. These findings may reflect the small but growing evidence base of the effectiveness of app-delivered gambling interventions as a potentially stand-alone support to reduce problem gambling severity, gambling symptom severity, gambling cravings, and gambling behaviour (Dowling et al., 2024a; Hawker et al., 2021; Rodda et al., 2024a; So et al., 2020, 2024). Furthermore, these findings are consistent with reports that people with different mental health presentations perceive app-delivered psychological interventions as potentially effective (Bakker et al., 2016; Batra et al., 2017; Bruhns et al., 2021, 2023; Chan & Honey, 2022).

### Scepticism and Technology-Related Threats

Two negatively-framed dimensions of the general acceptability of app-delivered gambling interventions were also examined, including: (1) scepticism about the long-term effectiveness, provision of professional support, ability to implement strategies in everyday life, or potential to increase isolation/loneliness; and (2) perceived technology-related threats to the provision of help in crisis situations, the ability to learn skills to manage everyday life, staying motivated, or understanding therapeutic concepts relative to therapist-delivered treatment.

Overall, the sample endorsed varying levels of scepticism toward app-delivered gambling interventions, whereby 48.6% of participants had higher scepticism, 17.3% were neutral, and 34.1% had lower scepticism. Greater gambling expenditure and gambling-related harms predicted greater scepticism, where gambling harms was also an independent predictor. These results appear intuitive, as people who spend more money on gambling and experience far-reaching harms may fear that their gambling is harder to treat using self-directed app interventions, which may exacerbate feelings of isolation and helplessness. In addition, these results are broadly consistent with documented barriers to help-seeking, including uncertainty, doubt, and mistrust toward gambling treatment (Bellringer et al., 2008; Kaufman et al., 2017; Rockloff & Schofield, 2004). Hence, future studies are needed to explore the long-term effectiveness of app-delivered interventions and the potential inclusion of app features to support implementation in everyday life (e.g., just-in-time approaches; Dowling et al., 2024a, b; Nahum-Shani et al., 2014; Rodda et al., 2024a, 2024b) and a sense of connection with others (e.g., group forums).

The majority of the sample (69.4%) also endorsed technology-related threats associated with app-delivered gambling interventions compared to therapist-delivered treatment. Furthermore, living in a rural/regional area independently predicted less perceived threats, and using high-intensity gambling support options independently predicted greater perceived threats. People living in regional/rural areas often face structural barriers to accessing treatment, such as a lack of clinicians or nearby treatment options (Gainsbury et al., 2014), which may explain their relative openness toward remotely delivered interventions. In contrast, people who have used high-intensity support options have shown a potential preference toward, and experience of, in-person services. While app-delivered gambling interventions are generally not designed to provide crisis support, several apps have been developed to provide tailored interventions to people in moments of need, which can be identified by functions within the app (e.g., ecological momentary assessments of high-risk situations such as gambling cravings; Dowling et al., 2024a, b; Hawker et al., 2021; Merkouris et al., 2020) and to incorporate features designed to maintain user motivation (e.g., in-app rewards/badges). Nevertheless, future studies may benefit from exploring ways to emulate the high-intensity nature of in-person services (e.g., access to a therapist via the app; Dowling et al., 2018, 2021; Merkouris et al., 2017) and/or to offer adjunctive human support to redress the perceived technology-related threats associated with app-delivered gambling interventions.

### The Use of App-Delivered Gambling Interventions

Overall, approximately 1 in 5 participants (20.8%) reported that they had ever used an app-delivered gambling intervention. Furthermore, young people and people with higher use of low-intensity and high-intensity supports, greater problem gambling severity, and greater gambling harms had higher odds of having used an app-delivered gambling intervention, where the use of high- and low-intensity support options were independent predictors. These findings appear intuitive, as young people are more adept at integrating technology into their healthcare (Balaskas et al., 2023), people who have accessed other supports have already overcome barriers to help-seeking and shown an openness to utilising supports (Loy et al., 2019), and people experiencing more severe problems and far-reaching harms from their gambling may be more likely to experience crises that propel them toward treatment (Bijker et al., 2022; Loy et al., 2019). Unexpectedly, however, none of the four dimensions of acceptability predicted the use of app-delivered gambling intervention, which suggests a potential translation gap between people’s attitudes towards app interventions and their actual use, as well as potential limitations of the measure of acceptability used in this study, discussed below.

### Implications

Overall, the findings support the general acceptability of app-delivered gambling interventions among people with lifetime gambling problems, which adds to the growing body of evidence that they may be an acceptable modality for delivering evidence-based treatment (Dowling et al., 2024b; Hawker et al., 2021; Humphrey et al., 2022; Rodda et al., 2024b; So et al., 2020). Importantly, however, acceptability did not translate into actual app use, which may partially reflect the lack of legitimate, evidence-based gambling help apps available in Australia (Ridley et al., 2020). This finding, however, may also indicate that the newly developed APOI is not a valid measure of app acceptability, although previous research using this validated measure has found significant associations between app acceptability and use (Bruhns et al., 2021, 2023). Finally, these findings highlight a need for more research to investigate ways to enhance gambling app engagement, relevance, and uptake, as well as the integration of apps into blended gambling treatment approaches (Rodda et al., 2019), where different app user groups may require different approaches. For example, app engagement may be improved among younger people by incorporating brief video-style content that mirror the format of social media content. The study findings indicate that invested parties ought to simultaneously promote the uptake, effectiveness, and benefits of anonymity of app-delivered gambling interventions, particularly among receptive audiences (e.g., young people, rural/regional residents, those with greater problem gambling severity), while also redressing scepticism and perceived technology-related threats among vulnerable subgroups (e.g., those with greater gambling expenditure and gambling-related harms).

### Study Limitations

The study findings ought to be interpreted in light of several limitations. Firstly, there are limited examinations of the general acceptability of app-delivered psychological interventions utilising validated measures (Schröder et al., 2015); hence, the need to adapt a closely related and validated measure of the acceptability of psychological online interventions for use in this study. Future research would therefore benefit from operationalising the construct of acceptability in the context of app-delivered psychological interventions to support a more uniform approach to its examination, as well as comparisons between app interventions. For example, Sekhon and colleagues (2017) proposed a new definition and theoretical framework of the acceptability of healthcare interventions as “a multi-faceted construct that reflects the extent to which people delivering or receiving a healthcare intervention consider it to be appropriate, based on anticipated or experienced cognitive and emotional responses to the intervention” (p.1; Sekhon et al., 2017), thereby extending Schroder et al.’s (2015) definition as a purely cognitive construct. Future research could test the application of this theoretical framework to the acceptability of app-delivered interventions via the development and validation of questionnaires or visual analogue rating scales that measure the proposed component constructs, as recommended by the study authors. Secondly, while the sample enabled an examination of acceptability among an unbiased group of people who have, and have not, used app-delivered gambling interventions, the low rate of app use may have limited the study’s power to detect effects in some analyses. The single item measures of app use, which combined the use of two Australian apps (“100-Day Challenge”, “Reset”) and any other app, may not have fully captured use, given the variety of gambling-related intervention apps available (e.g., financial tracking apps, online blocking apps, etc.). Future research would also benefit from a multi-item measure of app use that clearly defines different types of intervention apps available.

## Conclusion

This study constitutes the first examination of the general acceptability of app-delivered gambling interventions, as well as the predictors of acceptability and use among people with a lifetime gambling problem (i.e., potential end users), which forms a foundational piece of support for future research investigating ways to increase their uptake. Overall, the majority of the sample endorsed the general acceptability of app-delivered gambling interventions, yet only one in five participants had actually used one. Uptake could be improved by promoting the effectiveness and anonymity of evidence-based app-delivered gambling interventions, particularly among receptive audiences (young people, rural/regional residents, those with greater problem gambling severity), and redressing scepticism and perceived technology-related threats among vulnerable subgroups (those with greater gambling expenditure and gambling-related harms).

## Supplementary Information

Below is the link to the electronic supplementary material.

Supplementary file1

## Footnotes

1 References to “problem gambling” are in the context of the Problem Gambling Severity Index, which is a questionnaire used to measure indicators of behavioural dependence (behavioural, emotional, and cognitive symptoms) and adverse consequences from gambling. “Gambling harm” is a closely related but distinct construct, which only considers adverse consequences. While all efforts have been made to avoid stigmatising language (which focus on individual responsibility), the authorship team would like to explicitly recognise that harm from gambling is influenced by a complex range of factors, including social, cultural, policy, legislative, economic and environmental issues, as well as the design and marketing of gambling products.
