# Supplementary material for: The General Acceptability and Use of Smartphone App-Delivered Interventions for Gambling in Australia
Source: J Gambl Stud. 2025 Jan 9;41(2):593–613. doi: 10.1007/s10899-024-10373-9 (PMC12116850; doi:10.1007/s10899-024-10373-9)
Supplement: Supplementary file 1 — Supplementary file1 [file 10899_2024_10373_MOESM1_ESM.docx]

**SUPPLEMENTARY MATERIAL**

**Table S1. Multivariate regression analyses of the relationship between the demographic and gambling-related characteristics significantly related to the acceptability of app-delivered gambling interventions and the lifetime use of app-delivered gambling interventions in univariate analyses (n=173)**

|  | **B** | **SE** | **95% CI** | **β** | ***p-*value** |
| --- | --- | --- | --- | --- | --- |
| Scepticism and Risk Perception^a^ |  |  |  |  |  |
| Gambling expenditure | -0.00 | 0.00 | -0.00, 0.00 | -0.11 | .162 |
| **Gambling harms** | **-0.06** | **0.03** | **-0.12, -0.01** | **-0.17** | **.033*** |
| Technology-related Threats |  |  |  |  |  |
| **Rural/regional residence** | **1.54** | **0.59** | **0.38, 2.70** | **0.19** | **.010*** |
| **High-intensity support** | **-0.35** | **0.17** | **-0.68, -0.03** | **-0.16** | **.035*** |
|  | **OR** | **SE** | **95% CI** | **-** | ***p-*value** |
| Use of app-delivered gambling interventions |  |  |  |  |  |
| Age (years) | 0.99 | 0.02 | 0.96, 1.03 |  | .539 |
| **High-intensity support** | **1.82** | **0.38** | **1.21, 2.73** |  | **.004**** |
| **Low-intensity support** | **3.49** | **1.09** | **1.89, 6.44** |  | **<.000***** |
| Problem gambling severity | 1.03 | 0.04 | 0.96, 1.11 |  | .430 |
| Note. B = unstandardised beta; β = standardised beta; SE = standard error; 95% CI = 95% confidence interval; OR = odds ratio. ^a^n=172 as extreme gambling expenditure outliers were trimmed. Linear regressions were utilised to explore the acceptability of app-delivered gambling interventions and binary logistic regressions were utilised to explore the lifetime use of app-delivered gambling interventions. Variables that were significant at p<0.05 in univariate analyses were explored further in multivariate analyses; gambling harms was removed from multivariate analyses involving problem gambling severity due to multicollinearity concerns. Higher APOI scores indicate greater acceptability of app-delivered gambling interventions (hence, higher scores on the Scepticism and Perception of Risks and Technology-related Threat subscales indicate lower scepticism and lower threat, respectively). **Significant at *p*<.05*, <.01**, and <.001***.** | | | | | |
